# Myopericarditis in Systemic Lupus Erythematosus Unmasking a Concomitant Diagnosis of Marfan Syndrome: A Case Report

**DOI:** 10.1155/crrh/1055310

**Published:** 2026-07-30

**Authors:** Guilherme Cabrera, Gabriella Neves, Wellinghton Dias, Edson Duarte, Klinger Soares Faíco-Filho

**Affiliations:** ^1^ Departamento de Medicina, Disciplina de Clínica Médica e Medicina Laboratorial, Escola Paulista de Medicina (EPM), Universidade Federal de São Paulo (UNIFESP), São Paulo, State of São Paulo, Brazil, unifesp.br

**Keywords:** aortic aneurysm, case report, connective tissue disease, Marfan syndrome, myopericarditis, systemic lupus erythematosus

## Abstract

**Introduction:**

Cardiovascular complications represent a major source of morbidity and mortality in systemic lupus erythematosus (SLE). While pericarditis is common, acute myopericarditis leading to significant systolic dysfunction is a less frequent but potentially life‐threatening manifestation. The diagnostic evaluation can be complicated by overlapping clinical features and the presence of underlying, undiagnosed comorbidities. Hereditary connective tissue disorders, such as Marfan syndrome, present their own spectrum of cardiovascular risks, and their coexistence with autoimmune diseases like SLE is rare and poses significant diagnostic and therapeutic challenges.

**Case Presentation:**

We present the case of a 32‐year‐old woman with a long‐standing diagnosis of SLE who was admitted with new‐onset heart failure. Her symptoms included progressive dyspnea and pleuritic chest pain. The initial workup revealed active systemic inflammation, a newly reduced left ventricular ejection fraction of 44%, and a significant fusiform aneurysm of the ascending aorta (4.4 cm) associated with a bicuspid aortic valve. Cardiac magnetic resonance imaging was highly suggestive of active myopericarditis. While the initial findings were attributed to her lupus, the significant aortopathy in a young patient, combined with high myopia and a slender build, prompted further investigation. A formal clinical evaluation confirmed a diagnosis of Marfan syndrome based on the revised Ghent nosology. The patient responded favorably to intensified immunosuppression for her myopericarditis and was concurrently started on guideline‐directed medical therapy for her aortopathy.

**Conclusion:**

This case underscores the importance of maintaining a broad differential diagnosis in complex clinical presentations. The diagnosis of lupus myopericarditis was complicated and ultimately overshadowed by the unmasking of Marfan syndrome, a finding that fundamentally altered the patient’s long‐term prognosis and management strategy. This report highlights the critical need for a multidisciplinary approach and illustrates how a seemingly straightforward complication of a known disease can be the gateway to identifying a second, life‐altering diagnosis.

## 1. Introduction

Systemic lupus erythematosus (SLE) is a multisystem autoimmune disease with a heterogeneous clinical course, in which cardiovascular involvement represents a major cause of morbidity and mortality [1, 2]. Cardiac manifestations include pericarditis, myocarditis, Libman–Sacks endocarditis, coronary artery disease, and pulmonary hypertension. Among these, lupus myopericarditis—an inflammatory process involving both the myocardium and pericardium—can lead to acute heart failure, arrhythmias, and cardiogenic shock [3, 4].

The diagnosis of lupus myocarditis remains challenging because symptoms are often nonspecific and may overlap with other causes of cardiac dysfunction in SLE, such as hypertension‐related remodeling or accelerated atherosclerosis. In this context, cardiac magnetic resonance (CMR) has become a key noninvasive modality for detecting myocardial inflammation through the identification of edema and fibrosis [5].

We report the case of a young woman with established SLE presenting with acute myopericarditis, whose diagnostic workup unexpectedly revealed features consistent with Marfan syndrome, a hereditary connective tissue disorder caused by mutations in the FBN1 gene [6, 7]. The coexistence of these two distinct connective tissue diseases—one autoimmune and one genetic—is exceedingly rare and highlights the importance of reassessing the broader clinical picture when findings, such as a significant aortic aneurysm, appear disproportionate to the primary diagnosis.

## 2. Case Presentation

A 32‐year‐old woman with an established diagnosis of SLE since 2019, first diagnosed at 26 years of age, was admitted to our tertiary care center for the evaluation of progressive dyspnea and chest pain. Her lupus was characterized by cutaneous, articular, serosal, hematological, and renal (Class V lupus nephritis) involvement and had been well controlled on a maintenance regimen of hydroxychloroquine and azathioprine. She had completed secondary education, was currently unemployed, and reported no prior obstetric history.

Her medical history was also significant for systemic arterial hypertension, high myopia, a previously diagnosed bicuspid aortic valve with minimal aortic regurgitation documented on transthoracic echocardiography in 2021, at which time no measurements of the aortic root or ascending aorta were reported, and a chronic occlusion of the right subclavian artery attributed to thoracic outlet syndrome. Her family history was positive for unspecified autoimmune diseases but negative for premature cardiovascular events or known genetic syndromes.

The patient remained asymptomatic until January 2025, when she first noted dyspnea during intense physical exertion. Over the subsequent 8 months, her symptoms insidiously progressed to the point of limiting her activities of daily living. She also described a moderate, retrosternal, pleuritic chest pain and a two‐month history of night sweats. Upon presentation, she was hemodynamically stable but hypertensive (blood pressure up to 156/123 mmHg). Physical examination was notable for an asymmetry of upper limb pulses but revealed no cardiac murmurs or signs of systemic congestion.

An ambulatory transthoracic echocardiogram performed just prior to admission had revealed a new reduction in her left ventricular ejection fraction (LVEF) to 44% with diffuse hypokinesis, a significant decline from a study in 2021 that showed preserved function. Laboratory investigations on admission demonstrated active systemic disease, with elevated C‐reactive protein and erythrocyte sedimentation rate, leukopenia, normocytic anemia, markedly elevated NT‐proBNP, and a modest elevation in high‐sensitivity troponin T. The patient had a well‐established diagnosis of SLE since 2019, including biopsy‐proven Class V lupus nephritis characterized by immune‐complex glomerular deposits consistent with lupus membranous nephropathy. At the time of admission, persistent renal involvement was evidenced by subnephrotic proteinuria (protein‐to‐creatinine ratio 0.53), leukocyturia, and dysmorphic hematuria, despite preserved renal function. Serologic evaluation revealed a positive antinuclear antibody (ANA) titer of 1:1280 with a homogeneous pattern, positive anti‐Ro and anti–double‐stranded DNA antibodies, and hypocomplementemia, with reduced C3 and C4 levels, consistent with immunological activity of SLE. Retrospective application of the SLEDAI‐2K yielded a score of 19 points, indicating high disease activity. This score was derived from pericarditis, proteinuria, hematuria, pyuria, hypocomplementemia, positive anti–double‐stranded DNA antibodies, and leukopenia. Taken together, these findings were considered indicative of ongoing lupus disease activity.

To further delineate the etiology of her presentation, a comprehensive imaging assessment was undertaken. Contrast‐enhanced chest computed tomography (CT) (Figure 1) ruled out pulmonary embolism but revealed two relevant findings: a small pericardial effusion and a fusiform aneurysm of the ascending aorta, measuring 4.4 cm in maximal diameter. Three‐dimensional CT reconstruction provided additional anatomical detail, clearly demonstrating the extent of the ascending aortic aneurysm and its relationship with the aortic root (Figure 2).

**FIGURE 1:**
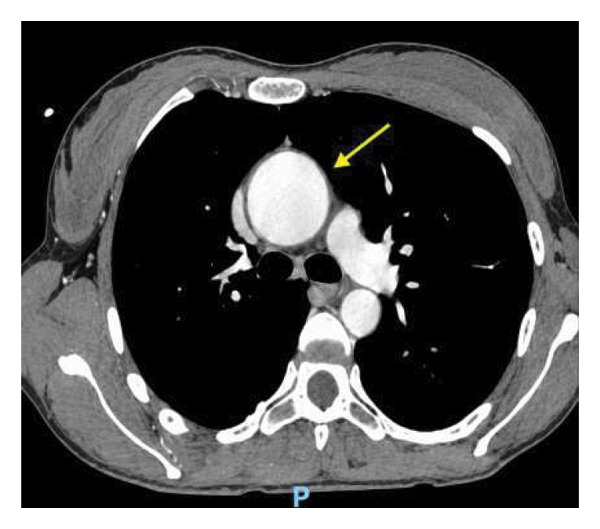
Contrast‐enhanced axial computed tomography pulmonary angiography (CTPA) demonstrating a fusiform aneurysm of the ascending aorta (arrow), measuring 4.4 cm in maximal diameter. No evidence of pulmonary embolism was identified.

**FIGURE 2:**
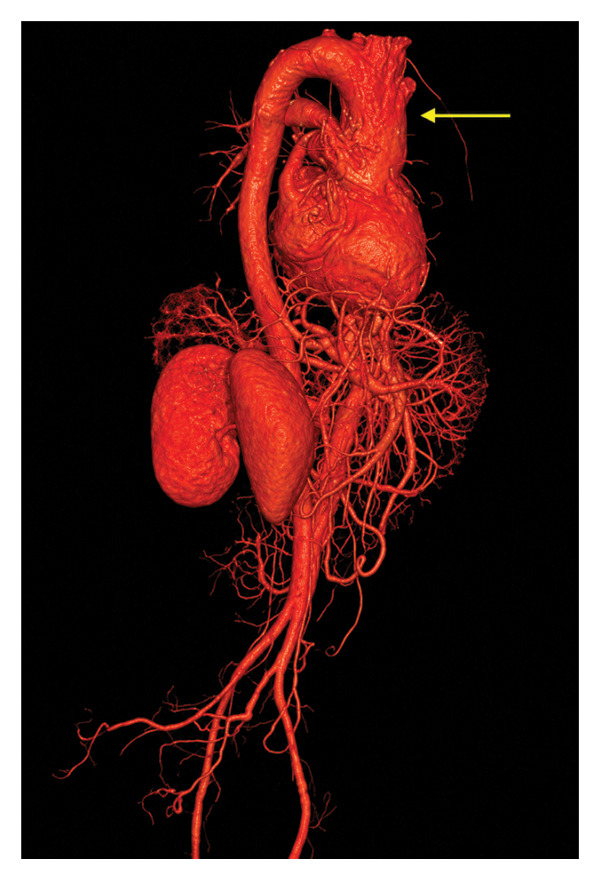
Three‐dimensional volume‐rendered computed tomography reconstruction of the thoracic aorta demonstrating fusiform dilatation of the ascending aorta (arrow), measuring 4.4 cm in maximal diameter.

Given the new onset of left ventricular dysfunction, coronary CT angiography was performed and demonstrated a calcium score of zero, with normal coronary anatomy and no evidence of significant obstructive disease, effectively excluding ischemic cardiomyopathy as the cause of ventricular dysfunction (Figure 3).

**FIGURE 3:**
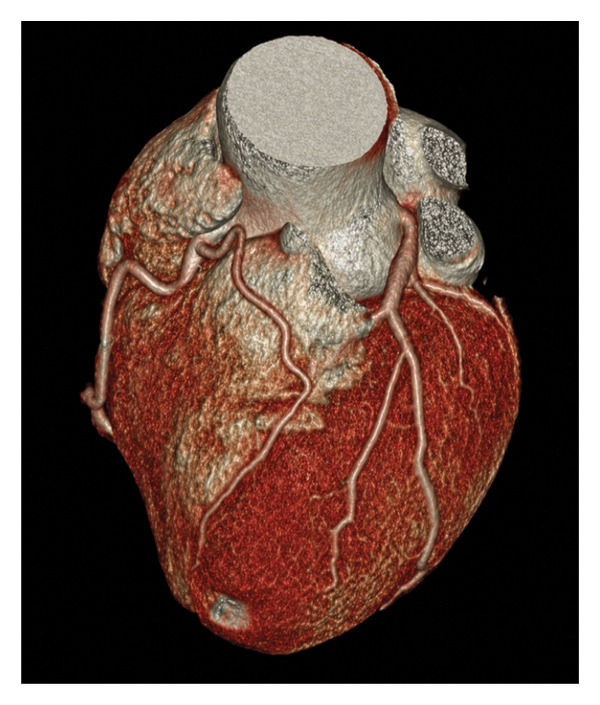
Three‐dimensional reconstruction from coronary computed tomography angiography (CCTA) demonstrating the absence of significant obstructive coronary artery disease. Coronary calcium score was zero, with no evidence of luminal stenosis.

CMR imaging constituted the cornerstone of the diagnostic evaluation. Cine steady‐state free precession sequences demonstrated mild left ventricular systolic dysfunction (LVEF 49%) with subtle hypokinesia of the apical septal segment (Figure 4). Tissue characterization showed late gadolinium enhancement (LGE) in a nonischemic, mid‐wall (mesocardial) distribution involving the mid‐apical inferoseptal segment, consistent with active myocardial inflammation (Figure 5). In addition, diffuse enhancement of the pericardial leaflets and a minimal pericardial effusion were observed, indicating concomitant pericardial involvement and supporting the diagnosis of acute myopericarditis (Figure 6). Although quantitative tissue characterization parameters such as T1/T2 mapping and extracellular volume (ECV) were not available, the overall CMR findings were considered highly suggestive of lupus myopericarditis in the appropriate clinical context.

**FIGURE 4:**
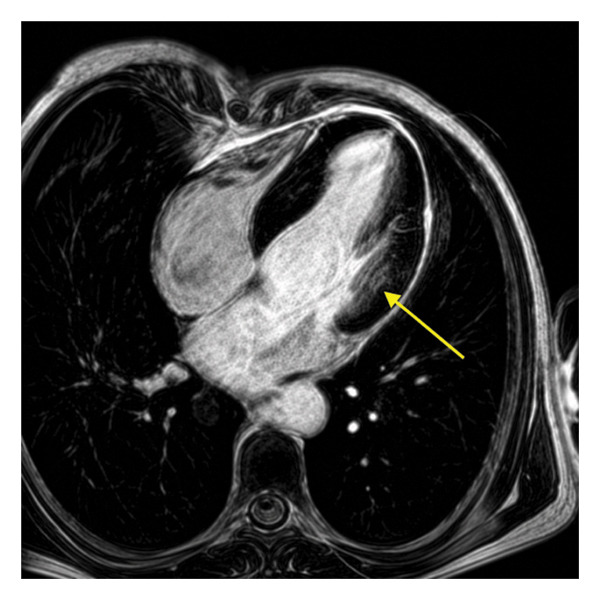
Cine steady‐state free precession (SSFP) cardiac magnetic resonance image demonstrating mild left ventricular systolic dysfunction. The arrow indicates the left ventricle, which exhibited a mildly reduced ejection fraction (49%) and subtle hypokinesia.

**FIGURE 5:**
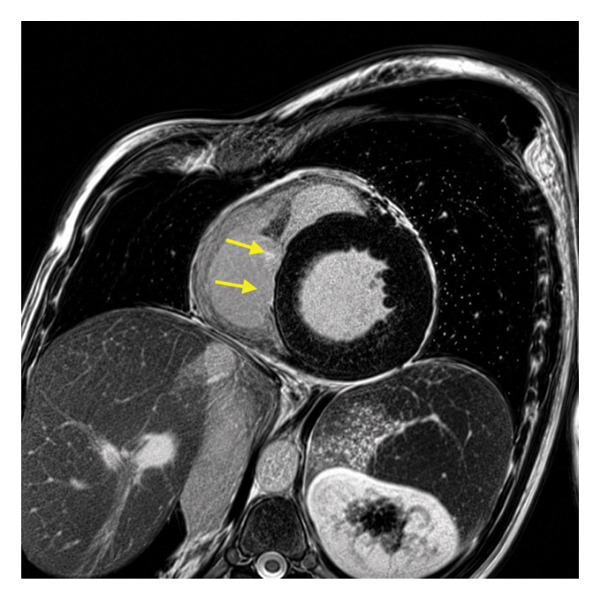
Cardiac magnetic resonance late gadolinium enhancement (LGE) sequence demonstrating a focal area of mid‐wall (mesocardial) enhancement in the mid‐apical inferoseptal segment of the left ventricle (arrows). This nonischemic enhancement pattern is consistent with myocardial inflammation and supports the diagnosis of myocarditis.

**FIGURE 6:**
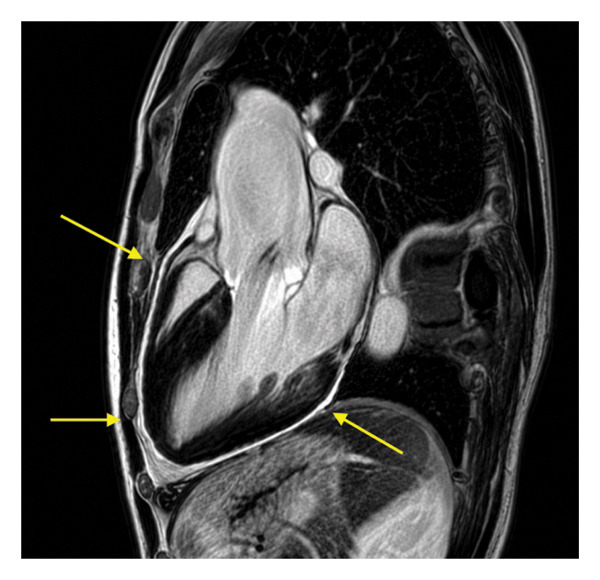
Cardiac magnetic resonance late gadolinium enhancement (LGE) sequence demonstrating enhancement of the pericardial leaflets (arrows), associated with a minimal pericardial effusion. These findings are consistent with active pericardial inflammation and support the diagnosis of acute pericarditis.

While the constellation of active systemic inflammation, new‐onset heart failure, and CMR findings strongly supported a diagnosis of acute lupus myopericarditis, the presence of a 4.4‐cm aortic aneurysm in a 32‐year‐old was a dissonant feature. Although vasculitis can rarely cause aortic aneurysms in SLE, the size of the aneurysm in this patient, coupled with her preexisting bicuspid aortic valve, high myopia, and slender body habitus, raised a strong suspicion for an underlying hereditary aortopathy. While the preexisting bicuspid aortic valve may have contributed to aortic enlargement, it did not fully account for the constellation of findings that ultimately fulfilled the revised Ghent criteria for Marfan syndrome. Further evaluation by the medical genetics team confirmed that the patient fulfilled the revised Ghent criteria for Marfan syndrome. She demonstrated significant aortic root dilatation (40 mm; Z‐score 4.21) and a systemic score of 8 points, derived from positive wrist and thumb signs (3 points), pectus excavatum (1 point), hindfoot deformity (2 points), scoliosis (1 point), and severe myopia greater than 3 diopters (1 point). Ophthalmologic examination revealed high myopia but no ectopia lentis. Although molecular testing for FBN1 variants had not been performed at the time of diagnosis, genetic counseling was initiated, and genetic testing is planned as part of her ongoing multidisciplinary follow‐up. Nevertheless, the combination of aortic root dilatation and a systemic score ≥ 7 was sufficient to establish the clinical diagnosis of Marfan syndrome according to the revised Ghent nosology. This new diagnosis fundamentally reframed her clinical picture from a single‐system complication of lupus to a complex interaction between two distinct connective tissue diseases.

Following the exclusion of an ischemic etiology and highly suggestive of myopericarditis, the patient was treated with intravenous methylprednisolone 500 mg daily for three consecutive days, followed by oral prednisone 60 mg/day with gradual outpatient tapering. Her maintenance immunosuppression was intensified by transitioning from azathioprine to mycophenolate mofetil 1500 mg twice daily. Concurrently, management for her newly diagnosed Marfan syndrome was initiated, carvedilol 3.125 mg twice daily and enalapril 20 mg twice daily to reduce aortic wall stress and optimize cardiovascular remodeling. Blood pressure control improved significantly following initiation of antihypertensive therapy and subsequently normalized during follow‐up. She responded promptly to immunosuppressive therapy with resolution of her chest pain and improvement in her dyspnea. At approximately 4‐month follow‐up, echocardiography demonstrated recovery of left ventricular systolic function, with improvement of the LVEF from 44% to 60%. At the same time, inflammatory markers and blood pressure had normalized, further supporting a favorable clinical response. NT‐proBNP levels decreased from 1904 to 429.7 pg/mL, troponin T decreased from 10 to 8 pg/mL, and inflammatory markers normalized (CRP from 30.9 to < 0.6 mg/L). Despite this recovery, beta‐blocker (carvedilol) and angiotensin‐converting enzyme inhibitor (enalapril) therapy were maintained because of the underlying Marfan‐associated aortopathy and the need for long‐term reduction of aortic wall stress. She was referred to a multidisciplinary team including specialists in rheumatology, cardiology, medical genetics, and cardiovascular surgery for long‐term surveillance and ongoing assessment of her Marfan‐associated aortopathy and future management strategies. Given that she represented the first recognized case of Marfan syndrome in her family, genetic counseling and cardiovascular screening of first‐degree relatives were incorporated into her longitudinal follow‐up plan.

The major clinical events, diagnostic investigations, therapeutic interventions, and follow‐up outcomes are summarized in Table 1.

**TABLE 1:** Timeline of key clinical events, diagnostic findings, and therapeutic interventions.

Date/period	Clinical event
2019	Diagnosis of systemic lupus erythematosus, including biopsy‐proven Class V lupus nephritis.
2021	Transthoracic echocardiography demonstrated preserved left ventricular systolic function (LVEF 68%) and a bicuspid aortic valve with minimal aortic regurgitation.
January 2025	Onset of exertional dyspnea.
Following months	Progressive worsening of dyspnea and development of pleuritic chest pain.
October 2025 (admission)	Hospital admission with heart failure symptoms, reduced LVEF (44%), and evidence of active systemic lupus erythematosus.
October 2025 (diagnostic imaging)	Cardiac MRI highly suggestive of myopericarditis and CT demonstrating ascending aortic aneurysm.
October 2025 (further evaluation)	Diagnosis of Marfan syndrome using the revised Ghent criteria.
October 2025 (treatment)	Treatment initiated with intravenous methylprednisolone, followed by oral prednisone, mycophenolate mofetil, carvedilol, and enalapril.
Follow‐up	Clinical improvement with recovery of left ventricular function and ongoing multidisciplinary follow‐up for both SLE and Marfan syndrome.

Abbreviations: CT, computed tomography; LVEF, left ventricular ejection fraction; MRI, magnetic resonance imaging; SLE, systemic lupus erythematosus.

## 3. Discussion

Lupus myopericarditis is an uncommon but potentially severe cardiac manifestation of SLE, often presenting with nonspecific symptoms and acute heart failure. Pericarditis affects approximately 16%–25% of patients with SLE, whereas myocarditis is less common, occurring in an estimated 3%–15% of cases. When myocardial involvement accompanies pericardial inflammation, clinically evident myopericarditis remains an infrequent presentation, reported in approximately 5.3% of patients with pericarditis. In such cases, CMR plays a crucial role by identifying nonischemic myocardial injury patterns compatible with inflammation. In this patient, the combination of heart failure symptoms, active systemic disease, and nonischemic LGE strongly supported the diagnosis of lupus myopericarditis. Alternative etiologies, including viral myocarditis, hypertensive cardiomyopathy, antiphospholipid syndrome, and SLE‐associated vasculitis, were considered during the diagnostic evaluation. However, the presence of active SLE, concomitant pericardial involvement, absence of significant coronary artery disease or thrombotic manifestations, and the marked clinical response to immunosuppressive therapy favored lupus myopericarditis as the most likely diagnosis. The favorable clinical response to intensified immunosuppressive therapy further reinforced this interpretation after exclusion of ischemic etiologies [4, 5, 8, 9].

The most distinctive feature of this case, however, was the unexpected identification of Marfan syndrome. The coexistence of SLE and Marfan syndrome is extremely rare. Given the estimated prevalence of Marfan syndrome (approximately 1 in 5000–15,000 individuals) and SLE (approximately 20–150 cases per 100,000 individuals), the probability of their coexistence by chance alone is exceedingly low. A review of the available literature identified very few reports describing patients with both conditions, highlighting the exceptional rarity of this association. In this context, the marked dilation of the ascending aorta represented a key finding that could not be adequately explained by SLE alone, prompting further investigation. Recognizing this additional diagnosis was essential, as the management strategies, genetic implications, and long‐term prognosis associated with Marfan syndrome differ significantly from those of SLE‐related cardiovascular manifestations [10–12].

The rarity of this association is biologically plausible, as the two diseases arise from fundamentally distinct pathogenic mechanisms. Marfan syndrome is an autosomal dominant connective tissue disorder caused by pathogenic variants in the FBN1 gene encoding fibrillin‐1, leading to extracellular matrix abnormalities and dysregulated transforming growth factor‐β (TGF‐β) signaling. In contrast, SLE is a multifactorial autoimmune disease characterized by loss of immune tolerance, autoantibody production, immune‐complex deposition, and chronic systemic inflammation. To date, no established biological mechanism has been identified that would predispose patients with Marfan syndrome to the development of SLE or other systemic autoimmune diseases. Therefore, their coexistence is currently regarded as most likely coincidental rather than the consequence of a shared pathogenic pathway.

Nevertheless, the simultaneous occurrence of these conditions may create important diagnostic challenges. Both diseases can affect multiple organ systems, including the cardiovascular, musculoskeletal, and cutaneous systems. Manifestations such as arthralgia, valvular abnormalities, serosal involvement, or cutaneous findings may complicate the diagnostic evaluation and potentially delay recognition of a second coexisting disorder. In the present case, the presence of a bicuspid aortic valve further complicated the interpretation of the aortic pathology, as bicuspid valve–associated aortopathy and other hereditary aortopathy syndromes were important differential diagnostic considerations. Nevertheless, the combination of significant aortic root dilatation and systemic manifestations fulfilling the revised Ghent criteria ultimately supported the diagnosis of Marfan syndrome, emphasizing the importance of maintaining a broad differential diagnosis when clinical findings cannot be fully explained by a single disease process.

This overlap also introduces therapeutic challenges. While lupus myopericarditis requires immunosuppressive treatment, prolonged corticosteroid exposure may adversely affect connective tissue and potentially contribute to aortic disease progression in patients with Marfan syndrome. In the present case, the diagnosis of Marfan syndrome did not alter the indication for immunosuppressive therapy, as prompt treatment of active lupus myopericarditis remained essential. However, awareness of the underlying hereditary aortopathy reinforced efforts to minimize long‐term corticosteroid exposure whenever clinically feasible and highlighted the importance of close cardiovascular surveillance. Therefore, multidisciplinary follow‐up is essential to balance autoimmune disease control with appropriate surveillance and management of aortic pathology. This case further illustrates the importance of reassessing the clinical picture when key findings are disproportionate to the primary diagnosis, as recognition of a concomitant condition may have substantial implications for prognosis, surveillance, and family counseling.

## Funding

The authors received no financial support for the research, authorship, and/or publication of this article.

## Ethics Statement

Ethical approval was not required for this study in accordance with local legislation and institutional requirements because this manuscript reports a single clinical case without experimental intervention. All procedures performed were part of routine clinical care and were conducted in accordance with the ethical standards of the institutional and national research committees and with the principles of the Declaration of Helsinki.

## Consent

Written informed consent was obtained from the patient for the publication of this case report and any accompanying images. The patient understands that the text and any pictures published in the article will be freely available on the internet and may be seen by the general public. The patient has been assured that her name and initials will not be published and that due effort will be made to conceal her identity.

## Conflicts of Interest

The authors declare no conflicts of interest.

## Patient Perspective

The patient expressed significant surprise at the diagnosis of Marfan syndrome, having attributed all her health issues to lupus for years. While initially overwhelmed by the prospect of managing a second chronic and serious condition, she conveyed gratitude for the thoroughness of the investigation that provided a complete picture of her health. She reported feeling empowered by this new understanding and expressed a strong commitment to the intensive, lifelong follow‐up plan, recognizing it as essential for her future well‐being.

## Data Availability

Data sharing is not applicable to this article as no datasets were generated or analyzed during the current study.
